# *In vivo* Analysis of CRISPR/Cas9 Induced Atlastin Pathological Mutations in *Drosophila*

**DOI:** 10.3389/fnins.2020.547746

**Published:** 2020-10-15

**Authors:** Aldo Montagna, Nicola Vajente, Diana Pendin, Andrea Daga

**Affiliations:** ^1^Laboratory of Molecular Biology, Scientific Institute IRCCS E. Medea, Lecco, Italy; ^2^Neuroscience Institute, Italian National Research Council, Padua, Italy; ^3^Department of Biomedical Sciences, University of Padua, Padua, Italy

**Keywords:** atlastin, mutation, CRISPR/Cas9, hereditary spastic paraplegia, endoplasmic reticulum, Golgi

## Abstract

The endoplasmic reticulum (ER) is a highly dynamic network whose shape is thought to be actively regulated by membrane resident proteins. Mutation of several such morphology regulators cause the neurological disorder Hereditary Sp astic Paraplegia (HSP), suggesting a critical role of ER shape maintenance in neuronal activity and function. Human Atlastin-1 mutations are responsible for SPG3A, the earliest onset and one of the more severe forms of dominant HSP. Atlastin has been initially identified in *Drosophila* as the GTPase responsible for the homotypic fusion of ER membrane. The majority of SPG3A-linked Atlastin-1 mutations map to the GTPase domain, potentially interfering with atlastin GTPase activity, and to the three-helix-bundle (3HB) domain, a region critical for homo-oligomerization. Here we have examined the *in vivo* effects of four pathogenetic missense mutations (two mapping to the GTPase domain and two to the 3HB domain) using two complementary approaches: CRISPR/Cas9 editing to introduce such variants in the endogenous atlastin gene and transgenesis to generate lines overexpressing atlastin carrying the same pathogenic variants. We found that all pathological mutations examined reduce atlastin activity *in vivo* although to different degrees of severity. Moreover, overexpression of the pathogenic variants in a wild type atlastin background does not give rise to the loss of function phenotypes expected for dominant negative mutations. These results indicate that the four pathological mutations investigated act through a loss of function mechanism.

## Introduction

Atlastin-1 is one of a three-member family of dynamin-like GTPases present in vertebrate genomes, however, single homologs of atlastin are present also in invertebrates, yeast and plants (Hu and Rapoport, 2016). The atlastins are membrane proteins, embedded in the endoplasmic reticulum (ER) bilayer, whose main function is to mediate ER homotypic membrane fusion, a process crucial for proper ER morphogenesis and maintenance (Hu and Rapoport, 2016). To date, however, this ability to promote fusion has been demonstrated exclusively for invertebrate atlastins (Orso et al., 2009; Anwar et al., 2012; Zhang et al., 2013; Wu et al., 2014). An involvement of atlastin in controlling Golgi morphology (Namekawa et al., 2007; Rismanchi et al., 2008; Chen et al., 2011; Behrendt et al., 2019) as well as in COPII formation (Niu et al., 2019) has also been proposed. However, the former was based exclusively on overexpression experiments and the latter has been identified using a very specific mutation (R48A/R77A) capable of uncoupling fusion and tethering (Pendin et al., 2011; Niu et al., 2019).

Mutations in Atlastin-1 have been identified as the cause of SPG3A, an autosomal dominant form of hereditary spastic paraplegia (HSP; Zhao et al., 2001; Zhao and Liu, 2017). The HSPs are a group of clinically heterogeneous neurological disorders classified into “pure” or “complicated” on the basis of the clinical features (Blackstone, 2018; Shribman et al., 2019). The pure HSP is defined by progressive spasticity and weakness limited to the lower limbs, while the complicated HSP may include other neurological manifestations (Blackstone, 2018). Clinically, HSPs can also be classified into early onset (1st decade of life) and late onset (between the 2nd and 4th decade) type. The main pathological changes associated with HSP include the axonal degeneration of the corticospinal tracts and back column (Shribman et al., 2019). There are currently over 80 genes or genetic loci linked to HSP (Blackstone, 2018). *Atlastin-1*-linked SPG3A is the second most common type of HSP accounting for approximately 10% of the autosomal dominant forms. More than 60 different *ATL1* gene mutations have been described, mostly missense mutations but also a limited number of small deletions, small insertions, splice site mutations, and whole exon deletions (Blackstone, 2018). Nevertheless, the genotype-phenotype correlation remains utterly unclear (Zhao and Liu, 2017).

Because of atlastins role in ER morphogenesis, the simplest explanation for the axonal degeneration associated with *SPG3A*-HSP would be the reduced ability of Atlastin-1/SPG3A disease variants to catalyze ER membrane fusion thus impairing network formation and/or maintenance. Although fascinating, the demonstration that a reduction in the membrane fusion activity of Atlastin-1 may be the reason for HSP lacks decisive experimental support. Indeed, while the fly atlastin (Orso et al., 2009) and other orthologs in more distantly related organisms (Anwar et al., 2012; Zhang et al., 2013) have been shown to produce *in vitro* the fusion of synthetic lipid bilayers, the human proteins do not display this ability. Thus, assessment of fusion has relied on reproducing a few conserved Atlastin-1 disease variants in the *Drosophila* ortholog and testing them for *in vitro* fusion (Bian et al., 2011). In addition, other pathological mutants were analyzed biochemically for their ability to hydrolyze GTP and dimerize in a nucleotide-dependent manner (Byrnes and Sondermann, 2011; O’Donnell et al., 2018). These studies, however, were unable to uncover an obvious correlation between disease-causing mutations and fusion/biochemical activity of atlastin.

It is furthermore uncertain whether ER morphology defects are the cause of *SPG3A*-HSP. The main reason for this is dual. Suitable animal models for the disease are unavailable and imaging the complex structure of the neuronal ER *in vivo* within the tissue in order to determine morphological differences between wild type and mutant ER is challenging if not impossible. In any case, further investigation is needed to determine whether *Atlastin-1*/*SPG3A* mutations consistently perturb ER network structure.

A functional replacement assay demonstrated that exogenously introduced *Drosophila* atlastin was capable of functionally replacing human Atlastins in HeLa cells depleted of the endogenous proteins (Faust et al., 2015). However, assessment of conserved pathological mutations in the *Drosophila* ortholog on both ER morphogenesis in HeLa cells and membrane fusion catalysis *in vitro* did not provide a deeper understanding of genotype-phenotype correlation (Ulengin et al., 2015). On the basis of these findings it has been questioned whether impaired ER membrane fusion is the exclusive driver of *SPG3A*-HSP.

To gain insight into the mechanism of *SPG3A*-HSP and verify the importance of ER morphology in disease pathophysiology, we examined the *in vivo* effects of four conserved pathological mutations introduced in the atlastin fly homolog. We took advantage of CRISPR/Cas9-mediated genome editing to introduce four different pathological mutations, including the most common R214C (R239C in humans), into the *Drosophila* atlastin gene. We reasoned that this approach would generate the closest possible system to a *SPG3A* patient where an endogenous allele is mutated.

## Materials and Methods

### *Drosophila* Stocks and Crosses

The UAS-Datlastin-myc and UAS-BiP-sfGFP-HDEL fly lines used in this study were described previously (Orso et al., 2009; Summerville et al., 2016). The Gal4 strains used were: tubulin-Gal4, elav-Gal4, D42-Gal4, and GMR-Gal4 obtained from Bloomington *Drosophila* Stock Center. To increase protein expression, experimental crosses were performed at 28°C, except for crosses with GMR-Gal4 which were performed at 25°C. Control genotypes included promoter-Gal4/+ individuals. Fly food was prepared using NUTRI-fly-IF mixture (Genesee Scientific), according to the manufacturer instructions.

### Constructs and Transgenic Lines Generation

*Drosophila atlastin* cDNA cloned in pCDNA3.1 in frame with a myc tag (Pendin et al., 2011) was mutagenized using Stratagene kit for site-directed mutagenesis. Primer pairs were designed as follows:

R192Q: 5′-AGTTCCTCGTCCAGGACTGGAGCTT-3′

R214C: 5′-ATTCTGAAACGATGTCTGGAGGTGT-3′

C350R: 5′-GCTCATGGAGGAGGTGCGCGGTGGAACGCG GC-3′

M383T: 5′-GCCAAGCGCAAGACGGGTGGTGAGGA GTTC-3′

Transgenic fly lines for the expression of atlastin carrying each of the mutations were generated by subcloning mutated myc-atlastin cDNA in pUAST vector. The plasmids obtained were microinjected in w^1118^ flies, thus generating transgenic lines UAS-atlastin^*R*192*Q*^, UAS-atlastin^*R*214*C*^, UAS-atlastin^*C*350*R*^, and UAS-atlastin^*M*383*T*^.

### Generation of CRISPR Mutants

CRISPR mutants were generated by microinjection (performed by BestGene Inc.) of two gRNAs, a 5′gRNA and a 3′gRNA, respectively, upstream and downstream of the atlastin gene fragment in the donor construct, cloned in the pCFD vector^1^, and a donor DNA template for homologous recombination into the y^2^ cho^2^ v^1^; attP40{nos-Cas9}/CyO strain (NIG-FLY stock #CAS-0001). Each donor DNA was constructed by Gibson assembly cloning in the BSSK(+) plasmid and contained a fragment of the *atlastin* gene carrying the desired pathological mutation and two flanking homology arms approximately 800 bp in length. The *atlastin* fragment was engineered to contain a novel *Eco*RI restriction site by silent mutagenesis for screening and identification of the mutant lines. Microinjected F_0_ flies were crossed *en masse* to TM3,Sb/TM6B,Tb flies (#2537, Bloomington *Drosophila* Stock Center), then F_1_ males were individually crossed to TM3,Sb/TM6B,Tb females. F_2_ sisters and brothers carrying TM6B balancers were crossed to obtain single lines. The presence of the correct mutation was identified by PCR followed by *Eco*RI restriction digest and confirmed by Sanger sequencing.

Primers:

For 5′gRNA: CTTCGTTGAGCACAATGCTGTCCT;

Rev 5′gRNA: AAACAGGACAGCATTGTGCTCAAC;

For 3′gRNA: CTTCGCAACTGGAAGATGATCTTG;

Rev 3′gRNA: AAACCAAGATCATCTTCCAGTTGC;

For 5′homology region: ctatagggcgaattgggtaccAAAAGGAAC AAATGAATAAGTG;

Rev 5′homology region: tgcactgaggACAGCATTGTGCT CAACG;

For atlastin: acaatgctgtCCTCAGTGCAGATATACAACC;

Rev atlastin: ttggtgaagaCCTCTTCAAGATCATCTTCC;

For 3′homology region: cttgaagaggTCTTCACCAACTAC CAAGC;

Rev 3′homology region: tggatcccccgggctgcaggGATGCCAAG TCAAGTTGC.

### Electron Microscopy

Larval brains were fixed in 4% paraformaldehyde and 2% glutaraldehyde and embedded as previously described (Orso et al., 2009). Electron microscopy (EM) images were acquired from thin sections under a FEI Tecnai-12 electron microscope at the DeBio imaging EM Facility (University of Padova). EM images of individual neurons for the measurement of the length of ER profiles were collected from three brains for each genotype. At least 40 neurons were analyzed for each genotype. Quantitative analyses of ER profiles length were performed with ImageJ software.

### Confocal Images of Larval Brains

Brains and ventral ganglia from third instar larvae expressing BiP-sfGFP-HDEL alone or together with mutated atlastin were dissected in M1 medium (30 mM HEPES, 150 mM NaCl, 5 mM KCl, 1 mM MgCl_2_, 35 mM sucrose, 5 glucose, and pH 7.2 at RT) containing 1 mM CaCl_2_. Motor neuron cell bodies from freshly dissected brains were imaged on a Leica TCS SP5 II confocal microscope, equipped with a HCX PL APO lambda blue 63×/1.40–0.60 Oil objective or a PlanApo 100×/1.4 Oil objective using an Argon laser line (488 nm). Confocal microscopy imaging was performed at 1024 × 1024 pixels per image, with a 200 Hz acquisition rate.

### HeLa Cells Transfection and Live Imaging

HeLa cells were cultured following standard protocols in DMEM (high glucose, from Merck) supplemented with fetal bovine serum (10%), L-glutamine (2 mM), penicillin (100 U/mL), and streptomycin (100 μg/mL). For fluorescence microscopy experiments, cells were plated on 18 mm coverslips and co-transfected when at 60% confluence with vectors for the expression of ER-sfGFP-3 (Addgene, plasmid #56482) and one of atlastin (wild type or mutated) plasmids (1 μg total DNA) using TransIT-LT1 transfection reagent (Mirus Bio LLC) according to the manufacturer procedures. 24 h after transfection, coverslips were mounted on an open top chamber thermostated at 37°C, covered with an extracellular-like medium (135 mM NaCl, 5 mM KCl, 1 mM MgCl_2_, 0.4 mM KH_2_PO_4_, 20 mM HEPES, 11 mM glucose, and pH 7.4 at 37°C) and imaged on a Leica TCS SP5 II confocal microscope, equipped with a HCX PL APO lambda blue 63×/1.40–0.60 Oil objective or a PlanApo 100×/1.4 Oil objective using the Argon laser line (488 nm). Confocal microscopy imaging was performed at 1024 × 1024 pixels per image, with a 200 Hz acquisition rate.

### Immunohistochemistry

CRISPR-mutations third-instar larvae raised at 25°C were harvested, dissected in PBS 1×, fixed in 4% paraformaldehyde for 15 min and washed twice in PBS containing 0.3% Triton X-100 (Sigma-Aldrich). Dissected larvae were probed with anti-GM130 (Abcam, ab30637, rabbit, 1:1000) overnight at 4°C, then washed 3 times with PBS plus 0.3% Triton X-100 and incubated with Alexa Fluor 555 anti-rabbit antibody (Molecular Probes Invitrogen, 1:500). Larvae were washed 3 times with PBS and mounted on coverslips using Mowiol (Sigma-Aldrich).

HeLa cells transfected as indicated above were fixed in PBS containing 4% paraformaldehyde for 15 min, incubated with 50 mM NH_4_Cl for 20 min, permeabilized with 0.1% Triton X-100 in PBS for 3 min and then blocked with 2% BSA and 0.2% gelatin for 30 min. Antibodies used were anti-GM130 (BD, #610822, mouse, 1:1000); anti-TGN46 (Abcam, ab50595, rabbit, 1:200). Alexa Fluor 555 conjugated goat anti-mouse and Alexa Fluor 647 conjugated goat anti-rabbit (Molecular Probes Invitrogen) were applied for 1 h at room temperature. Coverslips were mounted using Mowiol (Sigma-Aldrich).

All confocal images were acquired using a Leica TCS SP5 II confocal microscope, equipped with a PlanApo 100×/1.4 Oil objective, using a 543 nm laser line. Confocal microscopy imaging was performed at 1024 × 1024 pixels per image, with a 200 Hz acquisition rate.

### Protein Extraction and Western Blotting

Proteins were extracted from 20 fly heads expressing wild type or mutated atlastin under the control of the GMR-Gal4 promoter or from 20 brains extracted from CRISPR-mutant larvae. Heads or brains were placed in SDS-loading buffer, homogenized with a pestle and boiled for 5 min. Homogenates were cleared by centrifugation at 10,000 *g* for 5 min. Proteins were separated by SDS-PAGE, transferred into nitrocellulose membranes (GE Healthcare, 10600001) and probed using the following antibodies: anti-atlastin (Orso et al., 2009), 1:2000; anti-ACT (beta-actin; Sigma-Aldrich, A2228, 1:2500). The intensity of the bands was determined using Uvitec Alliance software (Uvitec Cambridge).

### Viability Assessment

For each CRISPR-mutation heterozygous flies were brother-sister crossed at 25°C. The offspring was collected and the number of individuals for each of the expected genotypic classes was counted. Results are expressed as the ratio of observed over expected percentage of individuals, normalized to a control experiment. At least 200 animals were counted from three independent crosses.

### Statistical Analyses

Data were analyzed using Microsoft Excel or GraphPad Prism 8. Average ER length values are expressed as mean ± standard error of the mean (SEM; *n* = number of profiles, unless otherwise specified). Statistical analyses were performed using unpaired Student’s *t*-test. Analyses of differences between fly populations were made using chi-square independent proportion analysis. Both tests were applied with a confidence interval of 95% (^∗^*p* < 0.05, ^∗∗^*p* < 0.01, and ^∗∗∗^*p* < 0.001).

## Results

While the basis for disease causation of mutations that disable atlastin GTP binding/hydrolysis activity seems to be rather evident, a large number of the variants examined shows only modest impairment of atlastin function when tested *in vitro*. To understand better the basis for atlastin-linked HSP, we took an *in vivo* approach in *Drosophila*. We selected four mutations: R192Q (corresponding to R217Q in Atlastin-1) defective in GTP hydrolysis, R214C (corresponding to R239C in Atlastin-1), C350R (corresponding to C375R in atlastin-1), and M383T (corresponding to M408T in Atlastin-1), both positioned in the 3HB region of atlastin. *In vitro* studies of these variants in atlastin-1 or Drosophila atlastin had shown very different behaviors. R192Q/R217Q is completely defective in dimerization, GTPase and fusion activities (Bian et al., 2011; Byrnes and Sondermann, 2011; Ulengin et al., 2015); R214C/R239C is essentially indistinguishable from wild type under all *in vitro* experimental paradigms (Byrnes and Sondermann, 2011; Ulengin et al., 2015), despite being the most common pathological mutation; the C350R/C375R variant has not been studied *in vitro* because it is insoluble due to protein folding or stability issues (Byrnes and Sondermann, 2011; Ulengin et al., 2015); M383T/M408T dimerization and GTPase activities have been shown to be slightly lower but comparable to wild type while its fusion activity has not been tested (Bian et al., 2011; Byrnes and Sondermann, 2011).

**FIGURE 1:**
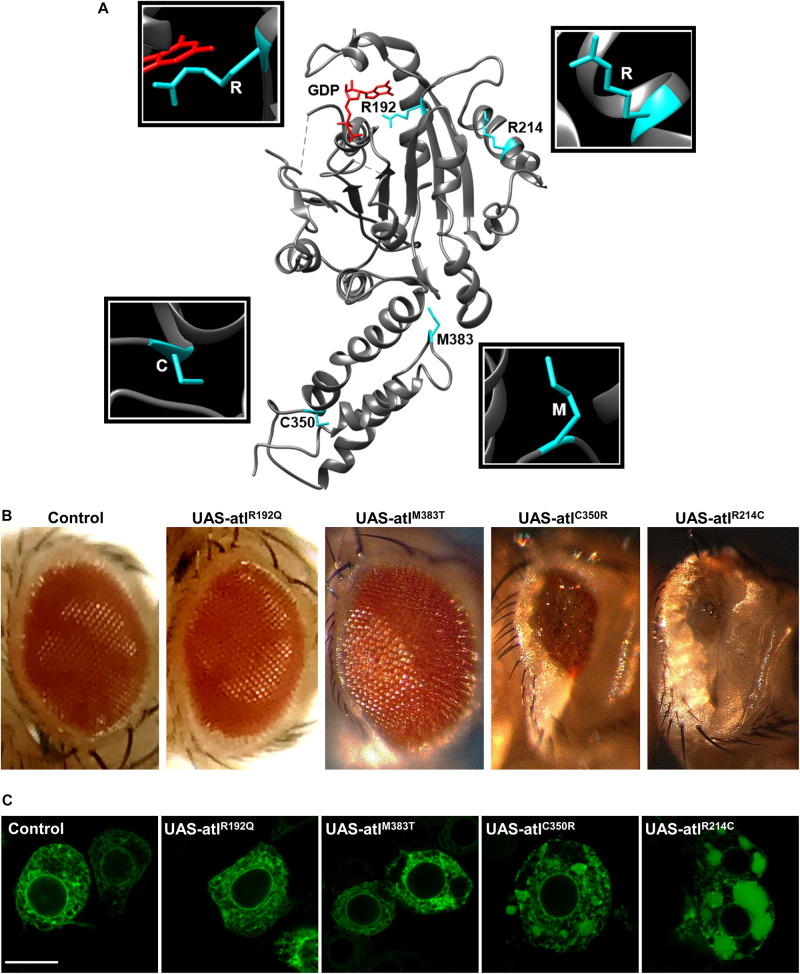
**(A)** Representation of atlastin protein structure (cytosolic domain only, PDB: 3X1D); the position of the mutated residues examined is highlighted. **(B)** Expression of UAS-atlastin carrying the indicated mutations driven by the GMR-Gal4 driver. **(C)** Confocal microscopy images of third instar larva brains co-expressing atlastin carrying the indicated mutations and the ER marker BiP-sfGFP-HDEL under the control of the motor neuron driver D42-Gal4. Scalebar 10 μm.

Transgenic lines for tissue specific expression of the selected pathogenic variants were generated to evaluate their severity in comparison to that of the wild type protein (Supplementary Figure 1A). *Drosophila* atlastin produces a small and rough eye when overexpressed under the control of the GMR-Gal4 driver and causes embryonic lethality upon ubiquitous overexpression with tub-Gal4 (Orso et al., 2009). Consistent with dysfunction in GTP hydrolysis, expression of UAS-atlastin^*R*192*Q*^ did not give rise to a rough eye nor did it cause lethality (Figure 1A). In contrast, expression of the other three variants in the fly eye resulted in a graded rough eye phenotype with UAS-atlastin^*R*214*C*^ yielding the most severe outcome and UAS-atlastin^*M*383*T*^ the mildest (Figure 1A). Upon ubiquitous overexpression, the R214C variant caused second/third larva stage lethality, suggesting a weak impairment of atlastin function, while overexpression of the C350R and M383T variants permitted adult eclosure indicating a more acute functional impairment of the protein. We conclude that all the pathological mutations examined reduce atlastin activity *in vivo* although to different degrees of severity.

To evaluate the impact of the overexpression of pathogenic variants of atlastin on ER morphology, we co-expressed in motor neurons the ER marker BiP-sfGFP-HDEL (Summerville et al., 2016) using the driver D42-Gal4 and examined third instar larva brains by confocal microscopy. Similar to the overexpression of wild type atlastin (Orso et al., 2009), the R214C mutant causes heavy overfusion of ER membranes with the resulting formation of large globular ER structures within the cytoplasm of neurons (Figure 1B). The size of these structures is progressively reduced concomitantly with the overexpression of variants that had weaker effects on eye morphology and lethality, confirming that the impact of these mutations on atlastin function *in vivo* increases along the series R214C, C350R, M383T, and R192Q (Figure 1B).

**FIGURE 2:**
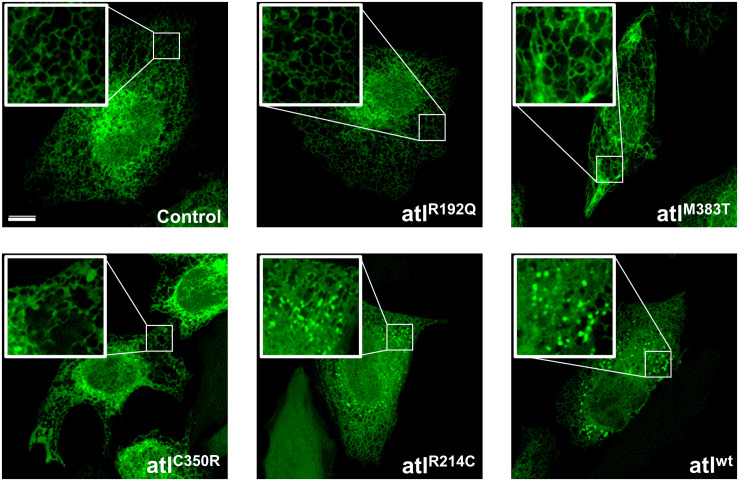
Confocal images of HeLa cells co-expressing UAS-atlastin carrying the indicated mutations and the luminal ER marker ER-sfGFP-3. Scalebar 10 μm.

To further build on this result, we studied how expression of *Drosophila* atlastin pathogenic mutants affects the morphology of the ER in HeLa cells where this organelle displays a flat, network-like shape. HeLa cells were thus co-transfected with individual atlastin mutations and the ER luminal marker sfGFP-ER-3. We found that, with the expected exception of the GTPase-dead R192Q mutant, expression of equivalent levels of the other variants disrupt ER morphology as indicated by the increased presence of bright fluorescence spots, indicative of ER hyperfusion, and by the progressive expansion of ER sheets and loss of ER tubules (Figure 2). In addition, overexpression of Drosophila wild type atlastin in HeLa cells induces dispersion of Golgi membranes (Pendin et al., 2011) and the pathological variants here analyzed also cause Golgi dispersion although less severely and in line with the observed gravity of their ER phenotypes (Supplementary Figure 2). These results indicate that in HeLa cells the behavior of atlastin pathogenic variants parallels that observed *in vivo* in flies, with the R192Q mutation being essentially inactive and R214C only barely less active than wild type atlastin.

Although the overexpression approach allows us to predict the severity of pathological mutations, it would be ideal to analyze their effects in a physiological context *in vivo*, by replacing one or two copies of the normal gene with the pathogenic variants so as to mimic the patients genetic condition. We therefore took advantage of the recently devised CRISPR/Cas9 technology to enable the introduction of the four mutant variants here studied in the endogenous *atlastin* gene. The four knock-in lines thus generated were named CRISPR-R192Q, CRISPR-R214C, CRISPR-C350R, and CRISPR-M383T. We initially established that the expression levels of mutant atlastin were comparable to those of wild type atlastin (Supplementary Figure 1B). On the basis of overexpression paradigms in cell culture (Rismanchi et al., 2008; Hu et al., 2009; Wang et al., 2016; Zhao et al., 2016) a dominant negative mechanism has been proposed for R217Q, a mutation that critically impacts the GTPase activity of Atlastin-1. We found that flies overexpressing UAS-atlastin^*R*192*Q*^ or flies heterozygous for CRISPR-R192Q are fully viable and do not display the lethality typically associated to atlastin loss of function, suggesting that this mutation in an *in vivo* model does not elicit a dominant negative effect. Likewise, heterozygous CRISPR-R214C, CRISPR-C350R, or CRISPR-M383T flies are viable indicating that, at least in *Drosophila*, these mutations do not function through a dominant negative mechanism.

**FIGURE 3:**
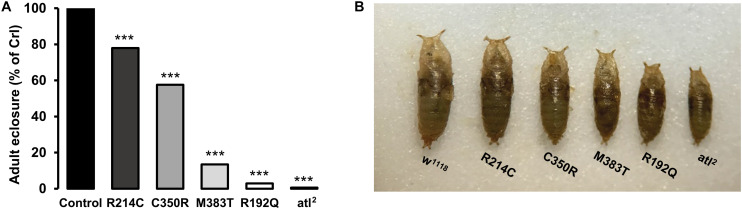
**(A)** Eclosion rates of homozygous CRISPR and *atl*^2^ mutants. The rate is calculated as the ratio between observed over expected individuals, normalized to controls. **(B)** Comparison of the size of homozygous CRISPR mutants with control (w^1118^) and *atl*^2^ mutant flies. ***p < 0.001.

We then evaluated for potential phenotypes flies carrying the above pathogenic variants in homozygosity. Atlastin null mutations are homozygous embryonic lethal with a small number of escapers [1% for *atl*^2^ (Lee et al., 2009)] (Figure 3A), and all developmental stages characteristically display a much smaller size than w^1118^ controls (Figure 3B). Similarly, CRISPR-R192Q homozygous mutant individuals eclosed at a very low rate (3%; Figure 3A), in agreement with this mutation disabling atlastin GTPase activity, and when compared to w^1118^ controls had an obviously smaller size that was, however, perceptibly bigger than *atl*^2^ null mutants (Figure 3B). The other homozygous pathological CRISPR mutants displayed greater eclosion rates and thus a less severe lethality as well as a progressively larger size than atlastin null and CRISPR-R192Q mutants, suggesting a partial loss of function mechanism (Figure 3B). It is interesting to note that both the viability and size of CRISPR pathological mutants are inversely proportional to the gravity of the mutation that, consistent with our previous observations based on overexpression, increases along the sequence CRISPR-R214C, CRISPR-C350R, CRISPR-M383T, CRISPR-R192Q (Figures 3A,B).

**FIGURE 4:**
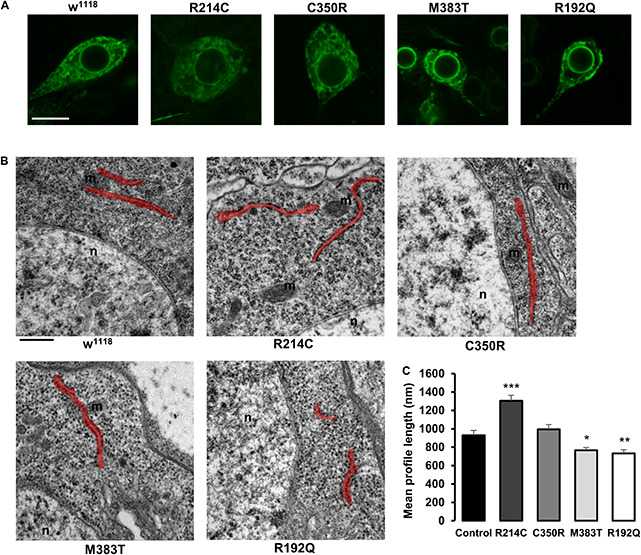
**(A)** Confocal microscopy images of third instar larva brain motor neurons of CRISPR mutants expressing the ER marker BiP-sfGFP-HDEL with the driver D42-Gal4. Scalebar 10 μm **(B)** TEM images of third instar larva brains neurons of CRISPR mutants. ER profiles are highlighted in red. Scalebar 500 nm **(C)** Quantification of the mean length of ER profiles measured in TEM sections. Mean ± SEM, n ≥ 50 profiles. *p < 0.05, **p < 0.01, ***p < 0.001.

To establish whether the presence of the CRISPRed pathological mutations had a bearing on ER morphology, we expressed the ER marker BiP-sfGFP-HDEL in mutant motor neurons and performed live confocal microscopy analysis. Because *Drosophila* third instar larva neurons are very small and embedded in living tissue, it turned out to be difficult to characterize in detail ER morphology, in order to define obvious differences in network organization among neurons expressing the different CRISPR mutations by fluorescence microscopy. Nevertheless, we observed that w^1118^ controls, homozygous R214C and homozygous C350R neurons displayed indistinguishable ER morphologies while R192Q and M383T had both altered ER shape (Figure 4A and Supplementary Figure 3). For this reason, we evaluated the length of ER profiles on thin EM sections of homozygous third instar larva mutant brains. ER profile length has been considered a parameter capable of reporting accurately on the functional state of atlastin since null *atl*^2^ mutant neurons are characterized by a much shorter average ER profile length than w^1118^ control neurons, a phenotype linked to increased ER fragmentation due to the prevalence of ER membrane fission in the absence of the fusion activity mediated by atlastin (Espadas et al., 2019). Our analysis reveals that the stronger the loss of function mutation, the shorter the neuronal ER profiles (Figures 4B,C). Indeed, CRISPR-R192Q displayed the shortest average ER profile length while the CRISPR-R214C variant had the longest (Figures 4B,C). Thus, the severity of the CRISPR homozygous mutants suggests a graded loss of function effect, confirming the results obtained from the previous experiments. We note that average ER profile length in controls (w^1118^ stock), while consistent with previous reports (Orso et al., 2009; Espadas et al., 2019; Vajente et al., 2019), is shorter than in some mutants as if mutants had a greater baseline profile length (Figures 4B,C). However, because the graded effect of the mutations is solid and all lines are grown under the same environmental conditions, we hypothesize that this could be due to the potentially very different genetic background between w^1118^ controls and mutants (Chandler et al., 2013).

**FIGURE 5:**
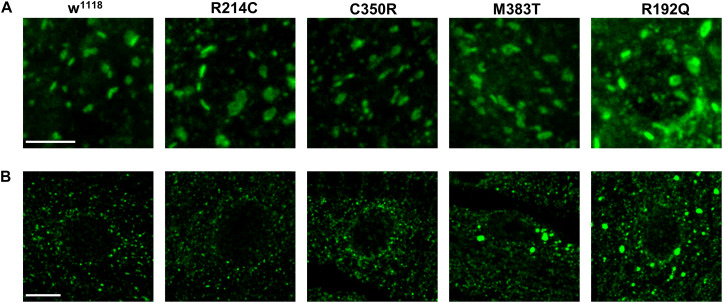
Confocal microscopy images of third instar larva motor neuron cell bodies **(A)** and body wall muscles **(B)** of CRISPR mutants, stained with *cis*-Golgi marker GM130. Scalebars: 5 μm in **(A)** and 10 μm in **(B)**.

**FIGURE 6:**
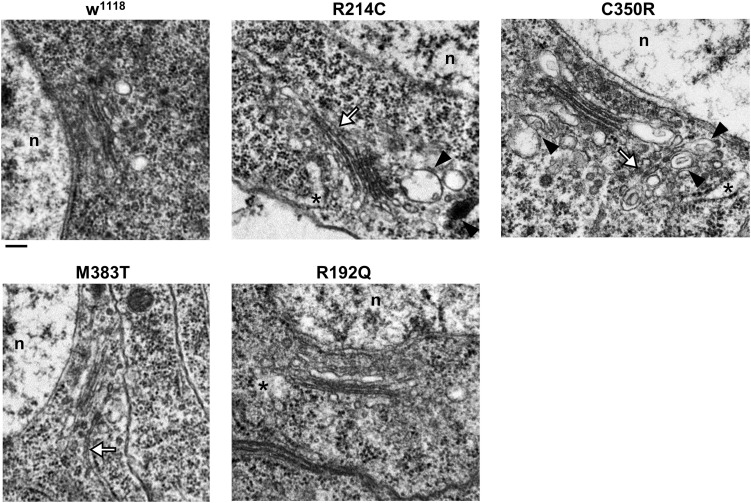
TEM images of third instar larva brains neurons of CRISPR mutants. Asterisks indicate dilated ER profiles; arrowheads indicate autophagosomes; white arrows indicate abnormally long Golgi cisternae/connecting two adjacent stacks. Scalebar 200 nm.

Several reports have suggested that expression of atlastin pathogenetic variants affects also Golgi apparatus morphology (Namekawa et al., 2007; Rismanchi et al., 2008; Chen et al., 2011; Behrendt et al., 2019). We thus investigated whether this was the case also in our knock-in CRISPR models. Immunohistochemical analysis of third instar larvae neuronal cell bodies and body wall muscles revealed that in all the mutant lines the structure and distribution of the Golgi apparatus were perturbed (Figure 5). When compared to w^1118^ controls, the Golgi stacks in knock-in mutants appear more abundant in the perinuclear region, where abnormally enlarged structures are occasionally present in the most severe mutants (Figure 5). Moreover, the number of dots corresponding to single stacks is increased in CRISPR larvae. When examined by TEM, the morphology of individual Golgi stacks looks altered, as dilated ER or Golgi cisternae are often present, as well as double- or multi-membrane autophagosomes (Figure 6). While in w^1118^ control flies, among 40 Golgi apparatus imaged, we found in only 17.5% of them dilated ER/Golgi cisternae and in 15% autophagosomes, mutant flies displayed more frequently these phenotypes (R192Q: 36% dilated Golgi, 19% autophagosomes; R214C: 41% dilated Golgi, 44% autophagosomes; C350R, 37% dilated Golgi, 29% autophagosomes; and M383T, 46% dilated Golgi, 19% autophagosomes).

## Discussion

Our study exploits *Drosophila* as an *in vivo* system to comprehend the role of organelle morphology in the onset of *SPG3A*-HSP disease and to model the mechanism whereby highly conserved pathological mutations cause this disease.

The morphological hallmark of the ER in atlastin null mutants is fragmentation, reported as a prominent shortening of the average length of neuronal ER profiles in EM sections. Pathological disease mutants when compared among themselves also display a progressive shortening of ER profiles that becomes more pronounced the more severe the mutation, according to the scale described in the above results. However, in all mutants the absolute value of ER profile length remains close to or greater than that of wild type ER. Irrespective of the underlying reason, this result suggests that ER morphology impairment may not be the prime driver of *SPG3A*-HSP pathology, as severe mutants die before reaching adulthood despite having an almost normal ER profile length, as measured by EM. Alternatively, it is possible that neuronal ER profile length as measured by EM is not an accurate readout for the complex morphological features of the ER. Although we have found evidence of morphological alterations within the Golgi apparatus of CRISPRed knock-in mutants, we believe that these alterations are too limited to be linked with the lethality of the mutants since it is known that integrity of the Golgi stacks is not required for proper secretory pathway trafficking (Kondylis and Rabouille, 2009; Dunlop et al., 2017). Our data suggest that more careful, deeper investigation of the function of affected organelles will be required to understand the root of this disease.

Although SPG3A mutations include small deletions, small insertions, splice site mutations, as well as whole exon deletions, the vast majority of them is represented by missense variants (Zhao and Liu, 2017). It has been suggested that a dominant negative mechanism underlies most of disease-causing mutation (O’Donnell et al., 2018), however, evidence for this supposition is largely based upon experiments conducted in cell culture upon overexpression of these variants. To reproduce a model more closely related to the pathological condition of patients, we introduced the mutations into the *Drosophila* genome by CRISPR/Cas9 editing in addition to examining their effects under overexpression *in vivo*. Four variants, two (R192Q and R214C) mapping to the GTPase domain of atlastin and the other two localized within the 3HB region (C350R and M383T), were studied. We found that the four pathological missense mutations investigated all act through a common loss of function mechanism though they differed for the severity of the phenotypes elicited. Indeed, all mutations in homozygosity caused a decrease of the adult eclosion rate and a reduction in size of all developmental stages, both typical features of *atlastin* null mutants. The gravity of the phenotypes, however, differed increasing along the succession R214C, C350R, M383T, R192Q such that the smaller R192Q homozygous individuals eclosed the least while the bigger R214C homozygous individuals eclosed at a greater rate. Corroboration of mutation severity came also from overexpression paradigms converging to show that the loss of atlastin function was almost complete for the GTPase-deficient R192Q and progressively decreased from M383T to C350R with R214C overexpression causing phenotypes almost as severe as those induced by wild type atlastin. Interestingly, we did not find evidence supporting the prevailing theory that most atlastin mutations act through a dominant negative mechanism (O’Donnell et al., 2018; Liu et al., 2019), not only because individuals heterozygous for all mutations eclose at normal rates and have normal size but also because overexpression of the pathological variants in a wild type background, i.e., in the presence of endogenous atlastin levels, does not give rise to the loss (or partial loss) of function phenotypes expected for dominant negative mutations.

Our results underscore that there is no obvious correlation between the effects of pathological mutations *in vivo* and the biochemical activity of atlastin *in vitro*. In fact, while R192Q/R217Q is a known GTPase-deficient mutation and therefore predicted to cause loss of protein function, the GTPase activity, homodimerization ability of the R214C/R239C and M383T/M408T variants as well as the membrane fusion competence of R214C are comparable to those of wild type atlastin (Bian et al., 2011; Ulengin et al., 2015; O’Donnell et al., 2018). Yet, these variants *in vivo* cause the same, though graded, loss of function phenotypes with the mildest mutation R214C/R239C being by and large the most common variant identified in SPG3A patients (Ulengin et al., 2015; Zhao and Liu, 2017). In conclusion, all four pathological mutations here investigated, irrespective of their position within the protein and often in contrast with data on their *in vitro* activity, cause loss of atlastin function *in vivo* in flies suggesting that this is the main mechanistic road to *SPG3A*-HSP disease.

## Data Availability Statement

The raw data supporting the conclusions of this article will be made available by the authors, without undue reservation.

## Author Contributions

AD and DP conceived the work, wrote the manuscript, and secured funding. AM, NV, and DP performed the experiments and analyzed the results. All authors contributed to the article and approved the submitted version.

## Conflict of Interest

The authors declare that the research was conducted in the absence of any commercial or financial relationships that could be construed as a potential conflict of interest.

## Footnotes

1 http://www.crisprflydesign.org/grna-expressionvectors/

## Supplementary Material

Supplementary Figure 1 **(A,B)** Representative western blot **(A)** and quantification, normalized to β-actin levels **(B)**, of the amount of atlastin in heads of individuals expressing the wild type protein or carrying the indicated mutations under the control of the GMR-Gal4 driver. Note that the transgenic line expressing wild type atlastin has been selected for its lower expression levels in order to permit survival of adult flies. Protein extracts from control flies (w^1118^ strain) are loaded for comparison with endogenous atlastin levels. Mean ± SEM, *n* = 3. **(C,D)** Representative western blot **(C)** and quantification, normalized to β-actin levels **(D)**, of atlastin in brains of CRISPR homozygous mutants, compared to w^1118^ controls. Mean ± SEM, *n* = 3.

Supplementary Figure 2 Confocal images of HeLa cells co-expressing UAS-atlastin carrying the indicated mutations and the luminal ER marker ER-sfGFP-3. Cells are immunostained with *cis*-Golgi marker GM130 and *trans*-Golgi marker TGN46. Scalebar 10 μm.

Supplementary Figure 3 Confocal microscopy images of third instar larva ventral ganglia of CRISPR mutants expressing the ER marker BiP-sfGFP-HDEL with the driver D42-Gal4. Scalebar 10 μm. Higher magnification images are reported in Figure 4A.
