# Supplementary figures and images for: In vivo Analysis of CRISPR/Cas9 Induced Atlastin Pathological Mutations in Drosophila
Source: Front Neurosci. 2020 Oct 15;14:547746. doi: 10.3389/fnins.2020.547746 (PMC7593789; doi:10.3389/fnins.2020.547746)

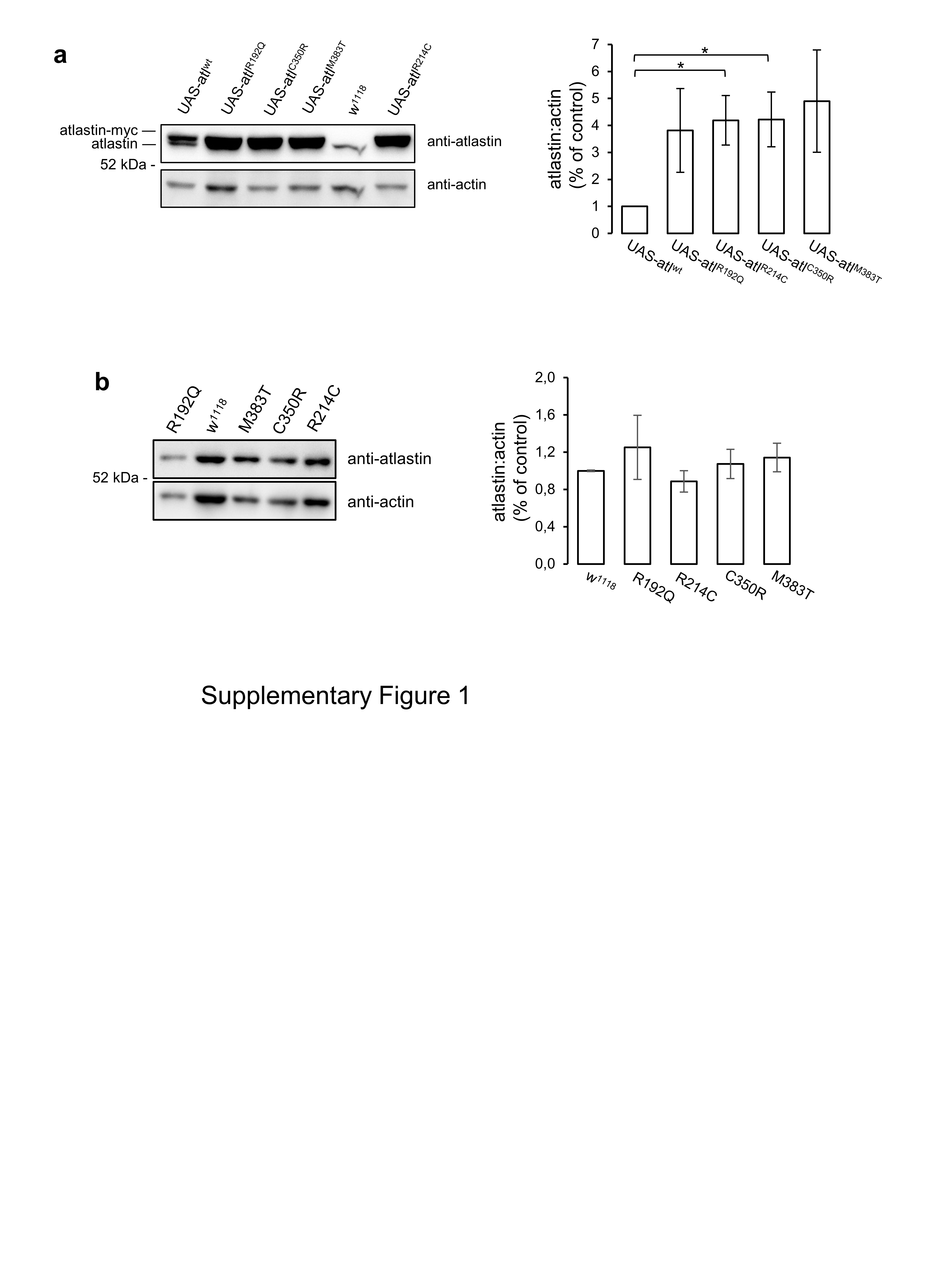

Supplement: Supplementary Figure 1 — (A,B) Representative western blot (A) and quantification, normalized to β-actin levels (B), of the amount of atlastin in heads of individuals expressing the wild type protein or carrying the indicated mutations under the control of the GMR-Gal4 driver. Note that the transgenic line expressing wild type atlastin has been selected for its lower expression levels in order to permit survival of adult flies. Protein extracts from control flies (w1118 strain) are loaded for comparison with endogenous atlastin levels. Mean ± SEM, n = 3. (C,D) Representative western blot (C) and quantification, normalized to β-actin levels (D), of atlastin in brains of CRISPR homozygous mutants, compared to w1118 controls. Mean ± SEM, n = 3. [file Image_1.TIF]

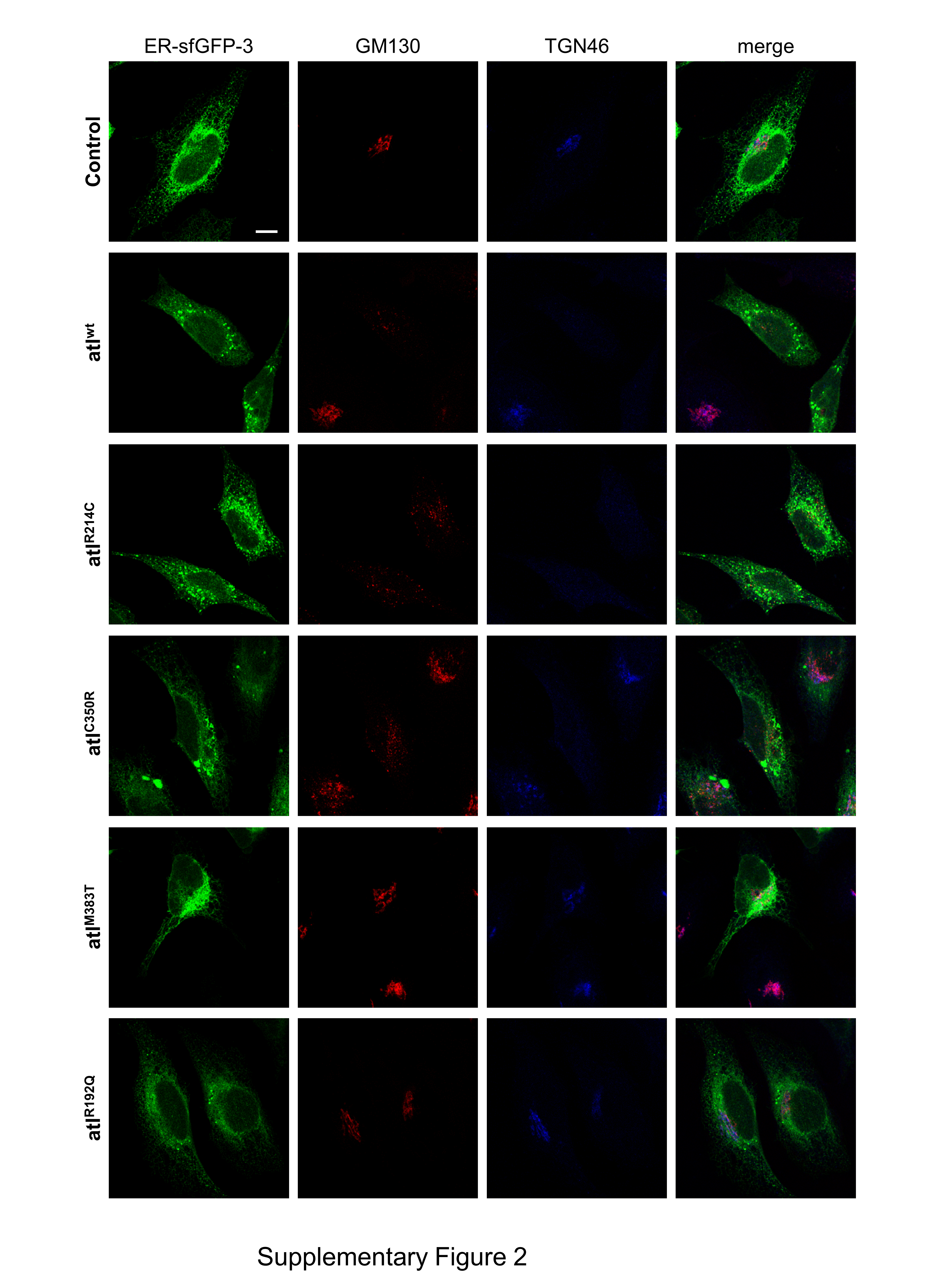

Supplement: Supplementary Figure 2 — Confocal images of HeLa cells co-expressing UAS-atlastin carrying the indicated mutations and the luminal ER marker ER-sfGFP-3. Cells are immunostained with cis-Golgi marker GM130 and trans-Golgi marker TGN46. Scalebar 10 μm. [file Image_2.TIF]

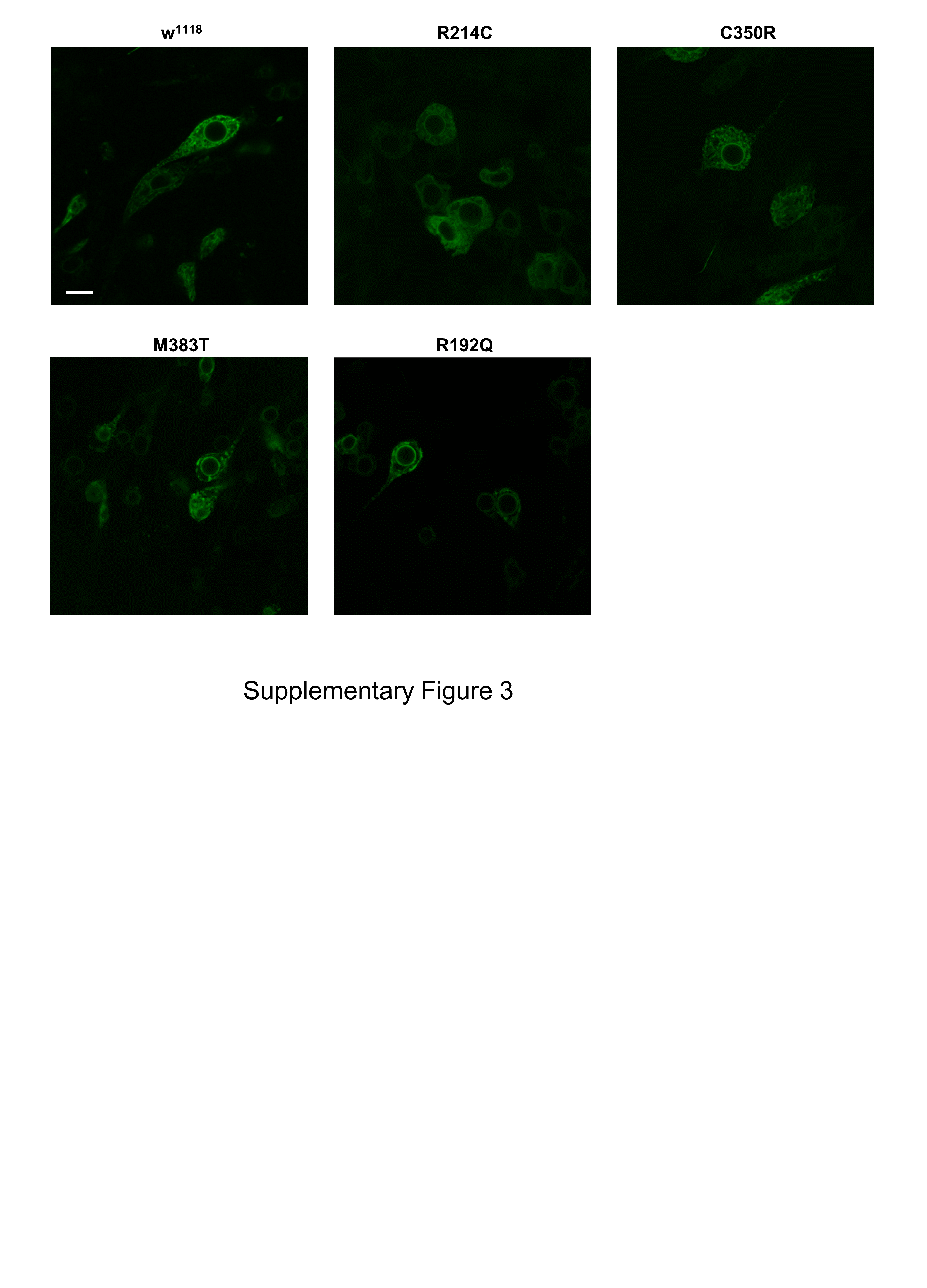

Supplement: Supplementary Figure 3 — Confocal microscopy images of third instar larva ventral ganglia of CRISPR mutants expressing the ER marker BiP-sfGFP-HDEL with the driver D42-Gal4. Scalebar 10 μm. Higher magnification images are reported in Figure 4A. [file Image_3.TIF]
